# The Impact of Ghrelin in Metabolic Diseases: An Immune Perspective

**DOI:** 10.1155/2017/4527980

**Published:** 2017-09-07

**Authors:** Jéssica Aparecida da Silva Pereira, Felipe Corrêa da Silva, Pedro Manoel Mendes de Moraes-Vieira

**Affiliations:** ^1^Laboratory of Immunometabolism, Department of Genetics, Evolution and Bioagents, Institute of Biology, University of Campinas, São Paulo, SP, Brazil; ^2^Department of Immunology, Institute of Biomedical Science, University of São Paulo, São Paulo, SP, Brazil

## Abstract

Obesity and insulin resistance have reached epidemic proportions. Obesogenic conditions are associated with increased risk for the development of other comorbidities and obesity-related diseases. In metabolic disorders, there is chronic low-grade inflammation induced by the activation of immune cells, especially in metabolic relevant organs such as white adipose tissue (WAT). These immune cells are regulated by environmental and systemic cues. Ghrelin is a peptide secreted mainly by X/A-like gastric cells and acts through the growth hormone secretagogue receptor (GHS-R). This receptor is broadly expressed in the central nervous system (CNS) and in several cell types, including immune cells. Studies show that ghrelin induces an orexigenic state, and there is increasing evidence implicating an immunoregulatory role for ghrelin. Ghrelin mainly acts on the innate and adaptive immune systems to suppress inflammation and induce an anti-inflammatory profile. In this review, we discuss the immunoregulatory roles of ghrelin, the mechanisms by which ghrelin acts and potential pharmacological applications for ghrelin in the treatment of obesity-associated inflammatory diseases, such as type 2 diabetes (T2D).

## 1. Introduction

The incidence of obesity and insulin resistance has increased in recent years. The World Health Organization (WHO) estimates that approximately 600 million adult people are obese [1]. Obesity directly impacts the economy and the quality of life of affected patients [2, 3]. Obesity is a disease with multifactorial origins and is characterized by excessive lipid accumulation in white adipose tissue (WAT), is promoted by the imbalance between caloric intake and energy expenditure [4, 5], and has harmful consequences to the individual [6]. Obesity is a risk factor for the development of other diseases, such as type 2 diabetes (T2D), metabolic syndrome, cardiovascular diseases, atherosclerosis, and several types of cancer [6–10].

WAT is a critical organ that contributes to host metabolism. Several cell types reside in WAT that regulate WAT and systemic homeostasis, such as adipocytes and preadipocytes, fibroblasts, macrophages, T lymphocytes, and among several other immune and nonimmune cells [11–15]. During obesity, immune alterations are observed in response to WAT expansion, which lead to a low-grade chronic inflammation. This inflammatory response is induced by changes in the recruitment of new leukocytes and also by changes in the function and activation status of adipose tissue resident macrophages (ATMs) and other leukocytes [6, 16, 17]. The immune changes that occur in WAT are characterized by the reduction of anti-inflammatory cytokines, such as interleukin 10 (IL-10), and upregulation of proinflammatory cytokines, such as tumor necrosis factor-*α* (TNF-*α*). This leads to the inhibition of the insulin-signaling pathway, which results in systemic insulin resistance [17].

Disruption of adipose tissue (AT) homeostasis and the induction of chronic systemic inflammation caused by obesity are complex processes and involve many players [16]. The disturbances within WAT microenvironment occur at the immune and metabolic levels in obesity and obesity-related conditions. Among these changes, increased levels of circulating free fatty acids (FFA) contribute to the development of insulin resistance [18]. Elevated levels of FFAs lead to the generation of new metabolites from FFA reesterification, such as diacylglycerol (DAG) [19]. DAG promotes the activation of several serine/threonine kinases, such as protein kinase C (PKC), which drastically impairs the phosphorylation of insulin receptor substrates (IRS) 1/2, thereby disrupting insulin signaling [18–20].

Another important players in the induction and control of AT inflammation are the Toll-like receptors (TLR), in particular, TLR4 [21, 22]. TLR activation leads to defective cellular function in all metabolically relevant organs, such as the liver, pancreas, and WAT [20–24]. This defect in cellular function results in immune cell activation and inflammation subsequently leading to resistance to key metabolic hormones such as insulin, leptin, and ghrelin [23–27].

Ghrelin is a peptide-hormone/cytokine widely distributed throughout the gastric mucosa made up of 28 amino acids and is mainly secreted by X/A-like enteroendocrine cells [28–31]. Ghrelin was described in 1999 as an endogenous ligand for the growth hormone secretagogue receptor (GHS-R) [32], a G-coupled receptor broadly expressed in the central nervous system (CNS) and in peripheral tissues, including nerve cells, cardiac cells, adipocytes, and immune cells [32–34].

Ghrelin has an important role in obesity and metabolic-related disorders. It is most known for its role in appetite regulation, acting directly on hypothalamic neurons responsible that were involved in feeding behavior [35]. Beyond this “classic” function, ghrelin is also an immunomodulatory hormone, providing new perspectives for its relevance in metabolic diseases [36, 37]. In obesogenic conditions, ghrelin levels are reduced with a concomitant induction of chronic low-grade inflammation [23, 38]. These data strongly suggest a role for ghrelin in obesity-related pathological conditions in establishing and maintaining “metabolic inflammation” and expand our knowledge of ghrelin beyond its role in the CNS. In this review, we will discuss the participation of ghrelin in immunomodulatory events, the impact of this regulation on metabolic disorders, and the mechanisms by which ghrelin acts.

## 2. Ghrelin Structure, Function, and Receptor

The GHS-R has two isoforms, GHS-R1a and GHS-R1b [39]. Only GHS-R1a triggers a signaling pathway, which is induced by the binding of ghrelin [39]. The lack of GHS-R1b isoform activity is attributed to the absence of a third intracellular loop, which prevents G protein coupling [39, 40]. There is evidence describing the interaction between ghrelin receptor and other G-coupled receptors, such as dopamine, serotonin and melanocortin receptors, and even GHS-R1b [39, 41]. These interactions lead to conformational changes in GHS-R, which impact GHS-R1a signaling [39–41].

Secreted ghrelin is found in two distinct forms in the bloodstream [42]. One is the desacyl (desoctanoyl) form (desacyl-ghrelin), which is more stable and has higher serum concentration levels compared to other ghrelin form [43–45]. Desacyl-ghrelin is suggested to be a non-GHS-R1a ligand form of ghrelin under physiological conditions [31, 43, 45]. Desacyl-ghrelin has cardioprotective effects [46–48]. However, its functional role and the receptor by which desacyl-ghrelin binds remain unknown [39, 49]. The other form of ghrelin is the acylated form (acyl-ghrelin), which undergoes a posttranslational modification on serine residue 3 [50]. This acylated form corresponds to approximately 20% of total circulating ghrelin and is responsible for the biological effects of ghrelin [51] and indicates that acylation of ghrelin is an important step for the biological activity of this peptide [51–53].

The posttranslational structural modification observed in the acylated form of ghrelin is attributed to an enzyme, discovered in 2008 by Yang and colleagues [54], which is called ghrelin-*O*-acyltransferase (GOAT). GOAT is responsible for the acylation of the preproghrelin before it is transported to the Golgi apparatus [42]. In the Golgi vesicle, proghrelin is proteolytically cleaved by the prohormone convertase 1/3 (PC 1/3) [31, 55, 56].

Acyl-ghrelin has a wide range of functions in several tissues. Acyl-ghrelin stimulates growth hormone secretion by the pituitary gland and activates the hypothalamic orexigenic axis [57]. Ghrelin serum levels are increased during caloric restriction [30]. In the hypothalamic orexigenic axis, ghrelin induces the secretion of neuropeptides, such as AgRP (agouti-related protein) and NPY (neuropeptide Y) [58], which leads to increased food consumption and reduced energy expenditure [53].

In addition to the direct effects of ghrelin on the CNS, ghrelin regulates gastrointestinal motility [59, 60], energy homeostasis [61], and the cardiovascular and reproductive systems [62]. Ghrelin also participates in the regulation of other adipokines, such as leptin, and modulates a broad number of immune functions [31, 63].

## 3. Ghrelin Signaling

GHS-R1a is widely distributed in different tissues [32–34], and its activation by acyl-ghrelin involves several signaling pathways [28–30]. The most studied cell type regarding the mechanisms of actions of acyl-ghrelin is hypothalamic neurons [64, 65]. In these cells, acyl-ghrelin relies on 5′ adenosine monophosphate-activated protein kinase (AMPK) [66–68]. Acyl-ghrelin AMPK-dependent signaling acts by two distinct mechanisms in hypothalamic neurons, in which intracellular calcium influx [69] and cytoplasmic nutrient sensors, such as AMPK [70] and mammalian target of rapamycin (mTOR), are the main targets triggered by the binding of acyl-ghrelin to GHS-R1a [71, 72].

AMPK activation leads to an inhibition of acetyl-CoA carboxylase (ACC) through posttranslational modifications [66]. The cellular outcome of this inhibition is increased mitochondrial metabolism due to consecutive activation of carnitine palmitoyltransferase 1 (CPT1) [73, 74]. Increased fatty acid oxidation leads to the generation of reactive oxygen species (ROS) and consequently stimulates uncoupling protein 2 (UCP2) [75]. These events induce the expression of orexigenic neuropeptides and consequently feeding behavior [75]. AMPK activation through GHS-R1a can also be mediated by calcium calmodulin-dependent protein kinase-kinase 2 (CAMKK2) in response to elevated intracellular calcium concentrations [76–78]. There is also evidence for a dependency on Sirtuin 1 and p53 during AMPK activation through GHS-R1a signaling [79].

The cytoplasmic nutrient sensor mTOR has a key function in hypothalamic energy homeostasis [80]. Several reports indicate that the effects of acyl-ghrelin are mediated by mTOR signaling pathway activation [72, 81, 82]. Activation of this machinery is responsible for the phosphorylation of several transcriptional factors, which are key elements in the orexigenic response, such as forkhead box protein O1 (FOXO-1) and cAMP response element-binding protein (CREB) [71, 83]. These data indicate that acyl-ghrelin signaling pathway in hypothalamic neurons is dependent on the signaling machinery of nutrient sensing.

In immune cells, the mechanisms of acyl-ghrelin signaling are poorly explored. Avallone and colleagues [84] show that ghrelin signaling in macrophages is dependent on AMPK activation and peroxisome proliferator-activated receptor gamma (PPAR*γ*) [84]. Both proteins have established anti-inflammatory roles [81–86]. Further studies are required to fully characterize acyl-ghrelin signaling in immune cells. The dependence of AMPK and PPAR*γ* for the immunoregulatory features of acyl-ghrelin is consistent with the current understanding of ghrelin signaling events in hypothalamic neurons as well as the cellular modifications that immune cells undergo during the induction of an anti-inflammatory phenotype [85–87].

### 3.1. Mechanisms Independent of Nutrient Sensors

There are two physiological outcomes as resultant of ghrelin receptor activation that does not depend on the nutrient sensing machinery: (i) the activation of hypothalamic neurons that evoke feeding behavior [69] and (ii) growth hormone (GH) secretion by pituitary cells [88]. Both outcomes are a direct result of elevated calcium levels, with distinct mechanisms of action. In the first case, activation of GHS-R1a leads to a subsequent elevation of cyclic adenosine monophosphate (cAMP), mediated by the adenilate cyclase (AC)-protein kinase A (PKA) signaling pathway [89]. As a result, hypothalamic neurons involved in feeding behavior are activated [69]. In pituitary cells, activated GHS-R1a induces calcium release from endoplasmic reticulum, which results in the activation of phospholipase C (PLC)-inositol triphosphate (IP_3_)-protein kinase C (PKC) pathway [39, 88].

## 4. Ghrelin, Obesity, and Inflammation

Tissue-secreted factors may disturb tissue homeostasis, which affects cellular and tissue metabolism and leads to systemic alterations [26]. Ghrelin and other factors regulate several aspects of metabolism and inflammation, which result in improved or worsened insulin resistance and metabolic syndrome [90–92]. Obesity-mediated metabolic disturbances increase levels of several cytokines and chemokines [91, 93]. This generates a proinflammatory status, which is a potential risk factor for the development of inflammation-induced insulin resistance [94]. One of these secreted factors is the monocyte chemotactic protein-1 (MCP-1), which is induced by the NF*κ*B pathway to recruit monocytes [95]. Because activation of GHS-R reduces NF*κ*B activation in endothelial cells [96], ghrelin treatment could limit immune cell activation through inhibition of NF*κ*B activation and subsequent MCP-1 secretion. This approach may lead to the development of new therapeutic approaches to treat T2D.

Metabolic imbalance induced by obesity leads to alterations in ATM population profile [97]. The M1 ATM macrophage population expresses the cell surface marker CD11c and secretes proinflammatory cytokines, such as tumor necrosis factor-*α* (TNF-*α*), interleukin-1*β* (IL-1*β*), interleukin-6 (IL-6), and monocyte chemotactic protein-1 (MCP-1) [98]. M1 macrophages also express high levels of iNOS (inducible nitric oxide synthase) [98, 99]. M2 macrophages express cell surface markers CD206, CD301, and CD163, secrete anti-inflammatory cytokines, such as interleukin-10 (IL-10), and express high levels of arginase-1 [15, 99]. M2 ATMs are often involved in homeostasis maintenance and tissue repair [15, 100]. The increase in the number of M1 ATM population in obesity is commonly accompanied with a reduction in M2 ATM population. This imbalance, with the predominance of a proinflammatory profile, inhibits the insulin-signaling pathway [94, 101, 102]. Thus, ATM function has an important role on metabolic syndrome and T2D development, which is frequently observed during obesity [99].

In obesogenic conditions, ghrelin levels are decreased [23] and levels of proinflammatory cytokines and adipokines, such as leptin, and liver-derived proteins, such as retinol binding 4 (RBP4), are increased [5, 103, 104]. Leptin is a proinflammatory adipokine, which inhibits ghrelin secretion [105] and worsens adipose tissue inflammation [5]. These data support the hypothesis that counter regulatory functions between leptin and ghrelin are an essential step for the maintenance of homeostasis CNS (food intake and energy expenditure regulation) and in immune responses [36].

## 5. Immunoregulatory Functions of Ghrelin

The wide distribution of functional ghrelin receptors (GHS-R) and their expression in various immune cell populations have attracted the attention of the scientific community. Changes in ghrelin levels can directly affect immune responses and tissue homeostasis [36, 37]. Leukocytes, such as adipose tissue macrophages (ATMs), express GHS-R [106] and detect changes in energy status [107, 108]. Thus, ghrelin actions on ATMs may play a role in the maintenance of the tissue homeostasis, suggesting a link between the immune system and systemic metabolism in response to different physiological and pathological conditions such as obesity and insulin resistance [109, 110].

**Figure 1:**
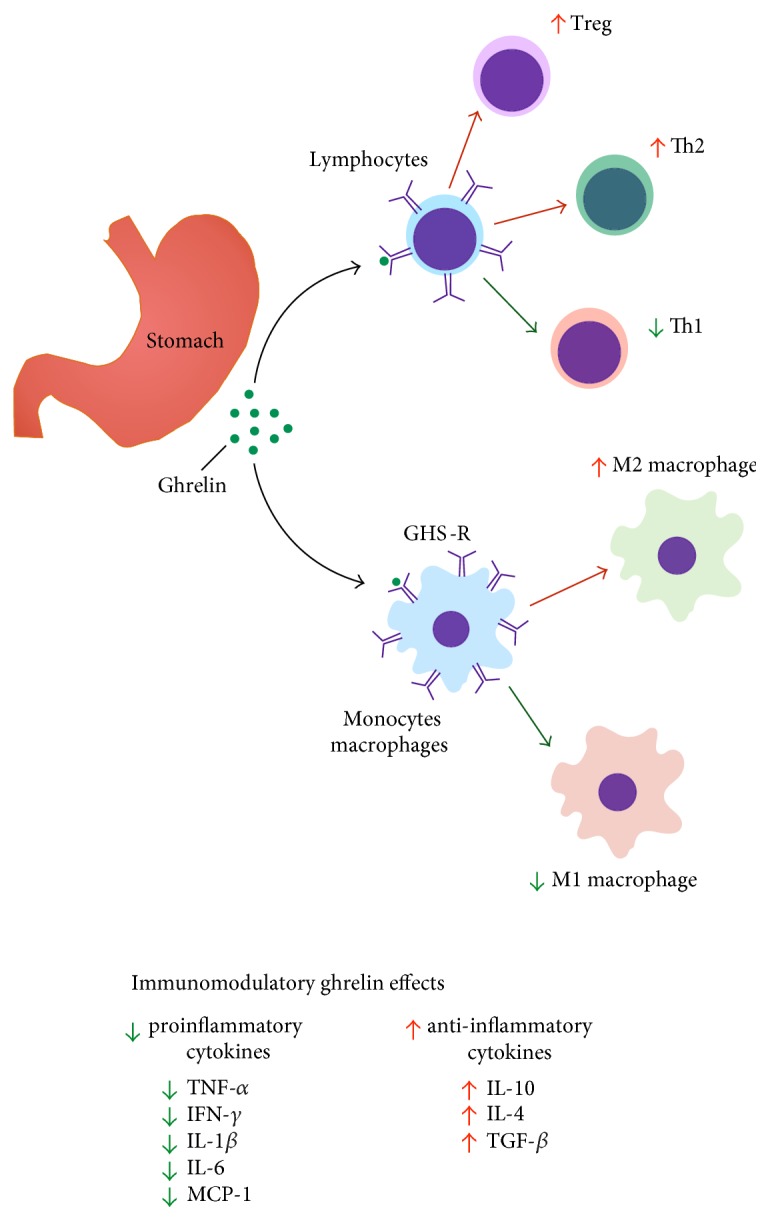
The immune roles of ghrelin. Ghrelin is secreted by X/A-like enteroendocrine cells. In the innate immune system, ghrelin acts on macrophages and induces an anti-inflammatory state (M2 profile) and inhibits proinflammatory macrophages (M1 profile). In the adaptive immune system, ghrelin exhibits an anti-inflammatory role. Ghrelin inhibits Th1 cells and increases the polarization of Th2 and regulatory T cells. These actions contribute to the reduced levels of proinflammatory cytokines and increased levels of anti-inflammatory cytokines.

Several studies support an immunoregulatory role for ghrelin [36, 37, 107, 111, 112]. Ghrelin acts on a diverse portfolio of leukocytes and directly alters immune cell function (Figure 1) [36, 37, 111, 112]. Dixit and colleagues [111] showed that ghrelin treatment *in vitro* inhibited the secretion of proinflammatory cytokines (IL-1*β*, IL-6, and TNF-*α*) in human monocytes, T cells, and peripheral blood mononuclear cells (PBMCs) [111]. Likewise, ghrelin treatment in human umbilical vein endothelial cells (HUVEC) reduced the secretion of IL-8 and MCP-1 and the activation of NF*κ*B in response to TNF-*α* stimuli [96]. Also, rats submitted to endotoxic shock with lipopolysaccharide (LPS) and treated with ghrelin had higher rates of survival compared to controls [113]. This protective effect was mediated by ghrelin-specific GHS-R receptor binding and resulted in reduced serum levels of TNF-*α*, IL-6, IL-8, and MCP-1 [96, 111]. Moreover, data suggests that mitogen-activated protein kinase phosphatase-1 (MKP-1) mediates the protective effect of ghrelin against endotoxic shock [114]. MKP-1 levels are reduced in inflammatory conditions, such as norepinephrine-induced sepsis, and lead to secretion of TNF-*α* [114]. Jacob and colleagues [114] reported that ghrelin treatment in septic rats increased gene and protein expression of MKP-1 [114]. This restoration of MKP-1 expression may partially explain the reduction in proinflammatory cytokines in response to ghrelin treatment. These data indicate that ghrelin can limit inflammation and plays an important role in metabolic and nonmetabolic inflammatory conditions.

*In vivo*, ghrelin has an anti-inflammatory and antinociceptive role [13, 34, 37, 111, 115–118]. Intraperitoneal administration of ghrelin in rats submitted to pain resulted in increased levels of serum IL-10 and TGF-*β* and reduced pain score [118]. The anti-inflammatory action of ghrelin was also observed in a colitis model. Ghrelin treatment reduced the expression of TNF-*α*, INF-*γ*, IL-1*α*, IL-1*β*, IL-6, IL-12, IL-15, IL-17, and IL-18 and increased IL-10 levels in colonic mucosa, which improved colitis score and survival rate in mice [37].

The anti-inflammatory roles of ghrelin can be extended to other inflammatory conditions, such as rheumatoid arthritis. Administration of the ghrelin agonist growth hormone-releasing peptide-2 (GHRP-2) reduced serum IL-6 levels and improved inflammation in arthritic rats [119]. Similar observations were obtained when peritoneal macrophages were treated with GHRP-2 *in vitro* [119]. Together, this data indicates that GHS-R can be used as a novel target for the treatment of acute and chronic inflammatory diseases.

Neutrophils play a fundamental role in immune response against pathogens and are regulated by ghrelin treatment. *In vivo* studies show that ghrelin treatment reduced neutrophil count in peritoneal lavage [63]. This was not attributed to enhanced apoptosis [120]. Neutrophils treated with ghrelin had increased phagocytic capacity and enhanced bactericidal capacity [120]. On the other hand, ghrelin treatment reduced neutrophil recruitment in the airways of subjects with chronic respiratory infections, which displayed reduced IL-8 and TNF-*α* levels in the sputum and improved overall inflammatory status [121].

Orlova and colleagues showed that ghrelin may affect dendritic cell- (DC-) mediated antigen presentation capacity. DCs treated with ghrelin had reduced capacity to induce the secretion of IL-17 and INF-*γ* and enhanced capacity to induce secretion of IL-10 and TGF-*β* from cocultured T cells [122]. Ghrelin also modulates thymic DCs. Ghrelin administration in the thymus induced the proliferation of DCs in aged mice [123], which contributes to a more effective maturation and response of effector and regulatory (Treg) T cell differentiation [124]. Ghrelin also regulates immune cell migration and proliferation. Ghrelin treatment reduced immune cell recruitment after LPS stimulation and induced the proliferation of Treg cells [34, 125]. Together, these data indicate that ghrelin has important effects in modulating T cells, especially regulatory T cells.

The role of adaptive immunity in the CNS is a growing topic of study. One of the most established models of the role of lymphocytes in CNS homeostasis is experimental autoimmune encephalomyelitis (EAE), an animal model of multiple sclerosis [126]. This condition has a marked proinflammatory feature, mediated by T CD4^+^ cells (Th1 and/or Th17 response) [125]. Ghrelin exerts therapeutic effects in EAE through the impairment of encephalitogenic Th1 and Th17 cells, and short-term ghrelin treatment reduces the clinical score of the disease [125]. This was associated with fewer infiltrated cells in the CNS and subsequent reduction in CNS inflammation. These effects were further improved by the induction of regulatory T cells in mice [125]. Souza-Moreira and colleagues [125] showed that ghrelin treatment suppressed M1 phenotype in microglia and reduced T cell infiltration, which was consistent with findings previously described by Theil and colleagues [125, 127]. The immunomodulatory roles of ghrelin in CNS are a result of decreased expression of proinflammatory cytokines, such as TNF-*α*, IL-1*β*, and IL-6, which indicate a noteworthy anti-inflammatory property.

## 6. Contrasting Roles of Ghrelin in the Immune System

There is evidence supporting the immunoregulatory role of acyl-ghrelin [107] and its beneficial effects to treat chronic inflammatory syndromes, especially acyl-ghrelin immunoprotective properties during endotoxic shock [36]. However, in sepsis, ghrelin may contribute to the higher mortality seen in septic mice [63]. This higher mortality was attributed to reduced neutrophil and natural killer cell activity, which led to increased bacterial burden [63]. Nevertheless, other authors demonstrated beneficial effects of acyl-ghrelin in the same experimental model and attributed these effects to lower inflammation in the hippocampus, observed by the reduction of TNF-*α* and IL-6 levels in septic brains [112].

Desacyl-ghrelin is a non-GHS-R1a ligand and was previously described as the nonactive form of ghrelin (although some authors report that desacyl-ghrelin binds to GHS-R1a when supraphysiological concentrations are induced) [34, 39, 44, 128]. Recent reports suggest that desacyl-ghrelin alters macrophage polarization *in vitro* [129]. Desacyl-ghrelin treatment decreased expression of TNF-*α* and CD11c and increased expression of CD206 in the mouse macrophage cell line (RAW264.7) [129]. Similar results were observed by the treatment of RAW cells with acyl-ghrelin [130]. This suggests that ghrelin treatment reduced M1 proinflammatory macrophage and increased M2 macrophage polarization [129]. These results are compatible with the observation that in humans, monocytes are the main targets for the anti-inflammatory actions of acyl-ghrelin [111]. However, the effects of acyl-ghrelin or desacyl-ghrelin treatment in bone marrow-derived macrophages or ATMs remain to be established.

Knockdown of GHS-R in mice reduced expression of TNF-*α*, IL-1*β*, IL-6, and MCP-1 in WAT of mice fed with a high-fructose corn syrup, which was associated with improved insulin sensitivity and obesity in aged mice. Also, ablation of the GHS-R promoted a shift towards a M2 profile in ATMs [106]. GHS-R ablation also limited the proinflammatory phenotype of peritoneal macrophages and ATMs, which was observed by decreased expression of proinflammatory cytokines [99].

Although a variety of studies indicates that ghrelin has an anti-inflammatory role, the controversial actions of this peptide support the need to better understand the mechanisms by which ghrelin acts on immune cells in response to different immunological challenges.

## 7. Ghrelin at the Neuroimmune Interface

### 7.1. Neuroprotective Effects of Ghrelin

In extrahypothalamic areas, acyl-ghrelin has a strong anti-inflammatory role [131–134]. Microglial cells express GHS-R, and acyl-ghrelin administration downregulates proinflammatory cytokine expression through impairment of microglial cell expansion [131–133]. The neuroprotective effects of acyl-ghrelin were extensively studied by many research groups [135–137]. In Parkinson's disease, there is a progressive neuronal degeneration of dopaminergic neurons localized in the *substantia nigra* and a concomitant increase in microglial activation. GHS-R1a is widely expressed in dopaminergic neurons in the *substantia nigra* [136, 137]. It is proposed that the neuroprotective effects of acyl-ghrelin in a Parkinson's disease context are partially due to the induction of tyrosine hydroxylase expression in dopaminergic neurons, a pivotal enzyme in dopamine biosynthesis [135]. The administration of MPTP (1-methyl-4-phenyl-1,2,3,6-tetrahydropyridine), a mitochondrial toxin, is a well-established model for Parkinson's disease studies, since there is a selective effect on dopaminergic neurons [138]. Mice treated with intraperitoneal acyl-ghrelin have lower levels of neuronal death and reduced dopamine loss [132, 133, 135]. Consistent with this phenotype, ghrelin treatment reduces proinflammatory markers, such as TNF-*α* and IL-1*β* [133]. It is proposed that the neuroprotective effects of ghrelin are also mediated through UCP2 activity [75, 135], since striatal neurons are dependent on UCP2 for optimal function [139–142].

Learning and memory retention also are influenced by acyl-ghrelin [143]. Intracerebroventricular [144] and intrahippocampal [145] acyl-ghrelin injections improve memory retention in rodents, which suggests a role for ghrelin in the molecular process of memory acquisition and/or consolidation. These results are reinforced by data published by Diano and colleagues [146], where spine density in the hippocampus is reduced in ghrelin knockout mice [146]. Data from Carlini and colleagues [144, 147] suggest that these beneficial effects are partially due to serotonergic inputs from dorsal raphe nucleus to the hippocampal circuits [147].

Several studies indicate that the brain is not a postmitotic structure in adult life [148–151]. Adult neurogenesis may be a potential therapeutic target for many neurodegenerative conditions [152–154]. The most explored structure in this context is the hippocampus [155]. There is evidence that acyl-ghrelin induces neurogenesis in brain structures related with cognition, such as the dendate gyrus of the hippocampus [156]. The classical target of ghrelin resides in the neuronal populations of the hypothalamus, and there are no reports to date describing the induction of neurogenesis by ghrelin. Therefore, neuroprotective effects of ghrelin in cognition-related structures may contribute to both the regulation of neurogenic events and the maintenance of mature resident cells [157–159].

The neuroprotective effects of ghrelin are also related to ischemic lesions, both *in vivo* and *in vitro* [160–162]. In these situations, there is insufficient blood flow into the brain. Treatment with acyl-ghrelin reduces ischemic lesions in mice by mechanisms both dependent and independent of GHS-R1a [160–162]. Here, neuroprotection is determined as reduced infarct tissue and cell death [161, 163].

### 7.2. Ghrelin, Stress, and Neuroinflammation

In view with the increased incidence of psychological disturbances and obesity, many groups have investigated the dynamic contribution of obesity to the development of affective disorders and how affective disorders affect obesity. Hormones that regulate energy homeostasis, such as ghrelin, may play a role in mediating psychological disturbances [164].

Serum levels of acyl- and desacyl-ghrelin, preproghrelin, and GH are increased in rodents submitted to acute and chronic stress models [164–168]. The hypothesis that increased ghrelin levels could be due to a stress response is supported by the involvement of ghrelin in neuroprotection, memory, and motivation [143]. GHS-R knockout mice have depressive behavior, which is marked by social isolation [165]. Cummings and colleagues showed that the increased ghrelin levels lead to decreased depressive behavior in rodents submitted to forced swimming test [165]. Psychological stress appears to induce inflammatory responses and is associated with compartmental alterations characterized by depressive symptoms [169]. Elevated levels of proinflammatory cytokines are found in patients with depression [170]. IL-1*β* and TNF-*α* increase serotonin uptake and metabolism, which contribute to depressive behavior. These data suggest a link between inflammatory responses and compartmental diseases [170]. GHS-R expression in the basolateral complex of the amygdala, an important region for emotional processing in rodents and humans, strongly supports the involvement of ghrelin in the modulation of emotional status and memory [171, 172]. Thus, ghrelin treatment could be an effective approach against emotional disorders due to ghrelin anti-inflammatory properties [143, 170].

Alterations in endogenous ghrelin levels and action could lead to the development of psychiatric disturbances associated to stress [172, 173]; a better understanding of how ghrelin regulates emotional behavioral disturbances is needed. These studies may contribute to the development of new targets for the treatment of diseases associated with stress and inflammation.

### 7.3. Ghrelin and Mediobasal Hypothalamus

The hypothalamus is a CNS structure primarily involved in global metabolic regulation [35]. There are multiple hypothalamic nuclei involved with metabolic regulation, such as the arcuate nucleus (Arc), lateral hypothalamic area (LHA), and paraventricular nucleus (PVN) [35]. The current model stipulates that Arc neuronal populations work in a binary-like system. The anorexigenic response is mediated by proopiomelanocortin (POMC) neurons, and the orexigenic response is mediated by agouti-related protein (AgRP) expressing neurons [35]. The signals induced by key metabolic hormones and nutrients are perpetuated by other neuronal populations in different hypothalamic nuclei that are synaptically connected to AgRP/POMC neurons [35]. Ghrelin exerts its orexigenic actions through AgRP neurons exclusively, since POMC neurons do not express GSH-R [174].

It is known that consumption of high-fat diet (HFD) leads to activation of proinflammatory processes in the hypothalamus with marked deregulation of feeding behavior and defective energy expenditure responses [175–177]. This inflammatory process is activated in the early stages of HFD feeding, with increased cytokine expression within 24 hours after HFD intake [176]. The key cell type that coordinates inflammatory responses in CNS is the microglia [178]. Several reports in the past decade described the importance of microglial cells in the initiation and perpetuation of hypothalamic inflammation and consequently its repercussions [175–177]. Following an obesogenic stimulus, microglial cells are activated in a TLR4-dependent manner and rapidly expand, creating a proinflammatory environment. These actions are restricted to the hypothalamus in the early stages of “metabolic inflammation” [175]. A recent report demonstrated that inhibition of microglial expansion in the Arc partially restored the metabolic impairments due to an obesogenic environment [177]. Thus, microglial activation is one of the most important targets for the development of focusing on the CNS under obese conditions.

The activation of TLR4-dependent responses in resident Arc microglial cells leads to a defective ghrelin orexigenic effect in animals fed with a chow diet [179]. This data indicates that microglia can directly modulate energy homeostasis by affecting neighboring cell functions and might explain the counterintuitive phenomenon of ghrelin resistance in obesity and metabolic diseases. However, ghrelin resistance is a complex subject of study and cannot be reduced to isolated effects on microglial or neuronal cells in the hypothalamus [180]. For instance, it is not known whether ghrelin resistance is due to (i) reduced ghrelin receptor expression/translocation in the target cells, (ii) defective ghrelin transport through blood-brain barrier as observed with leptin [181], or (iii) disrupted cellular homeostasis on ghrelin responsive cells, which is a similar process that leptin responsive cells undergo in obesogenic conditions [181, 182]. It is also possible that the physiological outcome is a combination of all of these factors. Another point to be clarified is whether neurons and glial cells are both resistant to the ghrelin signal and if there is temporal concordance in the establishment of ghrelin resistance in different cell types.

### 7.4. Obesity and Immunity in the Central Nervous System

**Figure 2:**
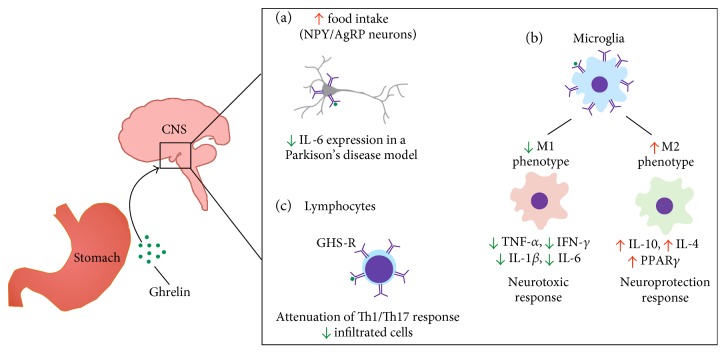
Ghrelin at the neuroimmune interface. Ghrelin has several functions in the CNS. (a) The classical orexigenic effect of ghrelin is mediated through activation of AgRP/NPY neurons in the mediobasal hypothalamus; it can also exert neuroprotective effects by diminishing IL-6 expression in striatal neurons in a Parkinson's disease model. (b) In microglia, ghrelin suppresses the proinflammatory phenotype and activates an anti-inflammatory program, which reinforces the neuroprotective role of ghrelin. (c) Infiltrated lymphoid cells are also decreased upon ghrelin treatment, with a marked reduction in Th1/Th17 responses.

The participation of Th1 or Th17 cells on hypothalamic impairment in metabolic diseases has yet to be clarified. It is known that T cells can penetrate the blood-brain barrier and act locally on the brain [183]. Thus far, there is no characterization of the participation of these cell types in the induction of metabolic inflammation on the hypothalamus. It is possible that ghrelin exerts an indirect immunomodulatory effect on adaptive immunity. As briefly discussed, Arc microglial activation can lead to an increase in local TNF [176]. This signaling has multiple effects and is one modulator of adaptive immunity in the periphery, which triggers adipose tissue lipolysis and raises triglyceride levels in the blood [184]. This in turn elevates the number of B cells and T cells with a Th1-type profile [184]. Thus, in obesity and obesity-related conditions, the immunomodulatory mechanisms of ghrelin have multiple points of actions and might not be restricted to the neural tissue (Figure 2).

Long-term studies indicate that bariatric surgery promotes a significant and sustained weight loss and recovery of metabolic parameters [185]. Several works have reported alterations in ghrelin levels during the postoperative period in patients submitted to different surgical strategies, which could be a result of altered body weight and feeding behavior [165, 186–206].

Faraj and colleagues [199] reported that ghrelin levels are dynamically modulated with weight loss [199] and patients with no changes in body weight do not have altered ghrelin levels. However, the role of ghrelin in postbariatric surgery is controversial [165, 186–206]. Besides, another relevant aspect that should be considered is the different methodological approaches used for the measurement of ghrelin, which could contribute to the discrepancy in ghrelin levels reported [207, 208].

Although the exact mechanisms by which bariatric surgery leads to reduced body weight are not completely understood, alterations in nutrient influx accompanied by increased ghrelin levels could lead to reduced proinflammatory marker expression, which will result in improved metabolic inflammation and the subsequent glucose homeostasis [199, 209–212].

## 8. Concluding Remarks

Ghrelin is not only a gastric peptide with CNS actions but it is also an important hormone/cytokine with important pleiotropic functions. The wide distribution of GHS-R1a in different cell types, including immune cells, indicates that ghrelin acts as a potent immunomodulator with powerful anti-inflammatory roles. The anti-inflammatory effects of ghrelin are observed in immune cells of both myeloid and lymphoid lineages. In macrophages/microglia, these anti-inflammatory properties are translated into increased secretion of anti-inflammatory cytokines, elevated M2/M1 ratio, and reduced proinflammatory cytokine expression. In lymphoid cells, ghrelin signaling leads to increased Th2 and Treg cell function. Therefore, ghrelin is a secreted hormone/cytokine with important anti-inflammatory roles in metabolically relevant organs, such as WAT and the hypothalamus. Ghrelin is a promising therapeutic strategy for the treatment of chronic inflammatory conditions, such as obesity. The therapeutic function of ghrelin is currently limited by its potent orexigenic properties. The paradoxical outcomes of ghrelin used to treat metabolic diseases are the induction of appetite and the anti-inflammatory roles. Thus, more studies are required to elucidate the molecular mechanisms of ghrelin actions as well as its application as a GHS-R agonist to treat obesity and insulin resistance in individuals.
